# Comparative study of phycoerythrobilin synthases for fine-tuning photosynthetic light-harvesting complexes, phycobilisomes

**DOI:** 10.1038/s41598-026-50582-3

**Published:** 2026-04-27

**Authors:** Mizuho Sato, Mai Watanabe, Misaki Iwata, Kaisei Maeda, Kaori Nimura-Matsune, Masahiko Ikeuchi, Rei Narikawa, Satoru Watanabe

**Affiliations:** 1 Department of Bioscience, Tokyo University of Agriculture, Tokyo, 156-8502 Japan; 2 Department of Biological Sciences, Graduate School of Science, Tokyo Metropolitan University, Tokyo, 192-0397 Japan; 3 Laboratory for Chemistry and Life Science, Institute of Integrated Research, Institute of Science Tokyo, Yokohama, 226-8503 Japan; 4 Graduate School of Arts and Sciences, University of Tokyo, Tokyo, 153-0041 Japan

**Keywords:** Phycobilisome, Cyanobacteria, Phycoerythrin, Phycocyanin, Photosynthesis, Biochemistry, Biophysics, Plant sciences

## Abstract

Phycobilisomes serve as the major light-harvesting antenna complexes in cyanobacteria; they capture light and transfer excitation energy to the photosystems, and their spectral and functional properties are primarily determined by the types of bilin chromophores attached to phycobiliproteins. We previously revealed that the properties of phycobilisomes can be modulated by altering the biosynthesis of these bilin chromophores. In this study, we introduced two distinct phycoerythrobilin (PEB) biosynthetic enzymes, *pcyX* and *pebS*, into the cyanobacterium *Synechococcus elongatus* PCC 7942 and successfully enabled PEB incorporation into native phycobilisomes. PebS exhibited high PEB synthesis activity comparable to the canonical PebA-PebB pathway, whereas PcyX showed lower activity, enabling fine-tuning of PEB accumulation. Consistent with previous findings, moderate PEB supply enhanced growth under green light; notably, PcyX expression further promoted growth by providing a more balanced pigment supply. Transcriptome analysis confirmed comparable expression of *pebA*-*pebB*, *pcyX*, and *pebS*, and revealed that increased PEB accumulation triggered broad metabolic remodeling: genes for glucose metabolism, hydrogenase complexes, potassium transporters, and regulatory factors including sigma factors were upregulated, suggesting a shift toward dissipation of excess reducing power. Together, these findings demonstrate that engineering bilin biosynthesis modifies light-harvesting properties and influences cellular metabolic balance, illustrating the potential of pigment pathway manipulation for redesigning photosynthetic function.

**Supplementary Information:**

The online version contains supplementary material available at 10.1038/s41598-026-50582-3.

## Introduction

Photosynthesis, the process by which light energy is converted into chemical energy, is fundamental to sustaining life on Earth. Throughout evolution, photosynthetic organisms have developed diverse and optimized light-harvesting complexes to efficiently capture light energy and transfer excitation energy to the photosynthetic reaction centers within their ecological niches. Phycobilisomes (PBSs), found in cyanobacteria, eukaryotic red algae, and glaucophytes, are peripheral light-harvesting complexes attached to the stromal side of the thylakoid membrane. PBSs enhance photosynthetic efficiency by capturing light in the green to orange spectral regions, which are poorly absorbed by chlorophyll, and transferring this energy to photosystems for conversion into chemical energy^1,2^.

Structurally, PBSs consist of rod and core subcomplexes composed mainly of phycobiliproteins and linker polypeptides. The rods comprise disk-like trimers, namely (αβ)_3_, which may further assemble as a hexamer ([αβ]_3_)_2_ composed of several types of phycobiliproteins, such as phycocyanins (PCs), phycoerythrins (PEs) and phycoerythrocyanins (PECs)^3–5^. The rods vary depending on the organism and can be made of PC only or a combination of PC and other phycobiliproteins such as PEs and PECs. The PBS core consists of a cylindrical structure composed of allophycocyanin (APC) with six PCBs per monomer. All units (disks within the rod and rod-to-core) are connected by linker proteins.

**Fig. 1:**
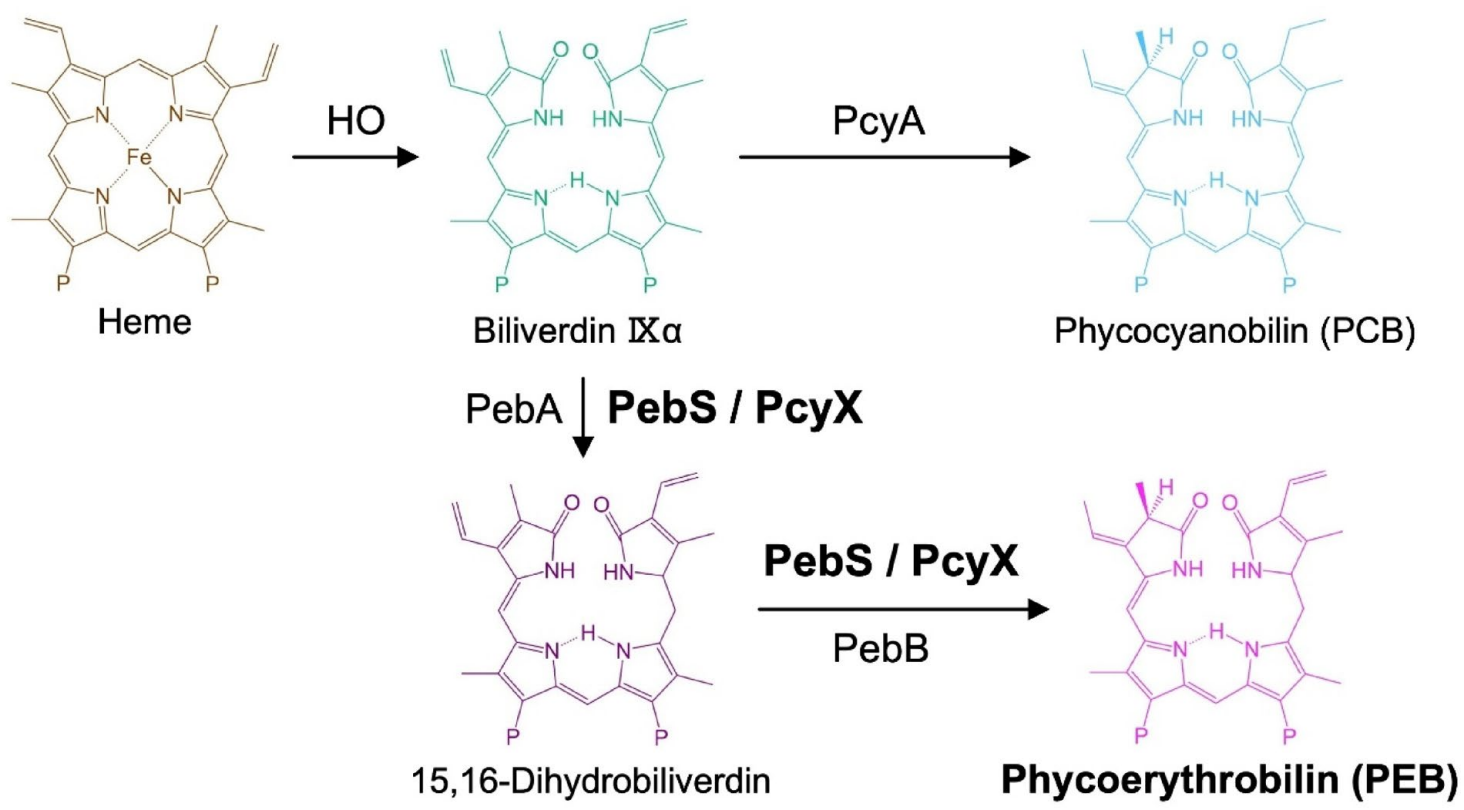
PCB and PEB metabolic pathways. Schematic overview of phycobilin biosynthesis from heme. In *Synechococcus* 7942, biliverdin IXα can be further converted to phycocyanobilin by the ferredoxin-dependent bilin reductase PcyA. Some cyanobacteria possess PebA and PebB in addition to PcyA, which convert biliverdin IXα to PEB in a two-step reaction. Meanwhile, PebS and PcyX, which are single-polypeptide ferredoxin-dependent bilin reductases (FDBRs) and have primarily been identified in cyanophage genomes and phage-derived metagenomic datasets, catalyze both reduction steps within a single polypeptide and are known to synthesize PEB independently via the 15,16-DHBV intermediate.

Each phycobiliprotein contains linear tetrapyrrole chromophores known as bilins that are covalently bound to cysteine residues within the apoproteins. The spectral properties of PBSs are primarily determined by the type and ratio of these bilins—such as phycocyanobilin (PCB), phycoerythrobilin (PEB), phycourobilin (PUB), and phycoviolobilin (PVB)—that define the wavelength of light absorption. The biosynthesis of bilins is catalyzed by ferredoxin-dependent bilin reductases (FDBRs), including PcyA, PebA, PebB, PebS, and PcyX^6–10^. PcyA catalyzes the conversion of biliverdin IXα to PCB, whereas PEB biosynthesis in cyanobacteria typically proceeds via the sequential action of PebA and PebB (Fig. 1). In contrast, PebS and PcyX are single-component FDBRs that catalyze both reduction steps within a single polypeptide (Fig. 1). Notably, these enzymes have primarily been identified in cyanophage genomes and phage-derived metagenomic datasets, highlighting the presence of alternative enzymatic strategies for chromophore biosynthesis are present in viral lineages infecting cyanobacteria.

Previous studies have demonstrated that the spectral properties of PBSs can be modified by engineering the biosynthetic pathways of bilins^11–13^. However, achieving precise control over bilin composition and incorporation remains challenging. In *Synechococcus elongatus* 7942 (*Synechococcus* 7942), heterologous production of phycoerythrobilin (PEB) has been reported to inhibit cell growth^13^. In cyanobacteria, bilins are known to participate in signal transduction by binding to bilin-binding photoreceptors known as cyanobacteriochromes (CBCRs) and bacteriophytochromes^14–16^. These photoreceptors contain a GAF domain that covalently binds bilins and mediate light sensing and downstream signal transduction. For instance, in *Synechococcus elongatus* UTEX 3055, PixJ, a bilin-binding photoreceptor, has been shown to regulate the expression of phototaxis-related genes^17^. Although *Synechococcus* 7942 lacks motility and does not exhibit phototaxis, introducing PEB biosynthesis into this strain, which natively lacks PEB, could potentially interfere with endogenous signaling networks. Nevertheless, the physiological consequences of such perturbations remain poorly understood.

In this study, we introduced PcyX and PebS into *Synechococcus* 7942 and systematically compared their contributions to PEB synthesis and PBS assembly. Transcriptome analysis revealed that the expression levels of the introduced *pebA*-*pebB*, *pcyX*, and *pebS* genes were comparable, yet the cellular responses to varying PEB accumulation differed. These included marked changes in genes related to glucose metabolism, hydrogenase complexes, potassium transporters, and regulatory factors such as sigma factors and circadian rhythm components, suggesting that cyanobacterial cells actively adjust redox balance and metabolic flow in response to bilin levels. Collectively, our findings reveal distinct activities between PcyX and PebS and demonstrate how these differences can be exploited to modulate light-harvesting capacity and promote growth under green-light conditions.

## Results

### Construction of cyanobacterial strains with controlled PEB levels

**Fig. 2:**
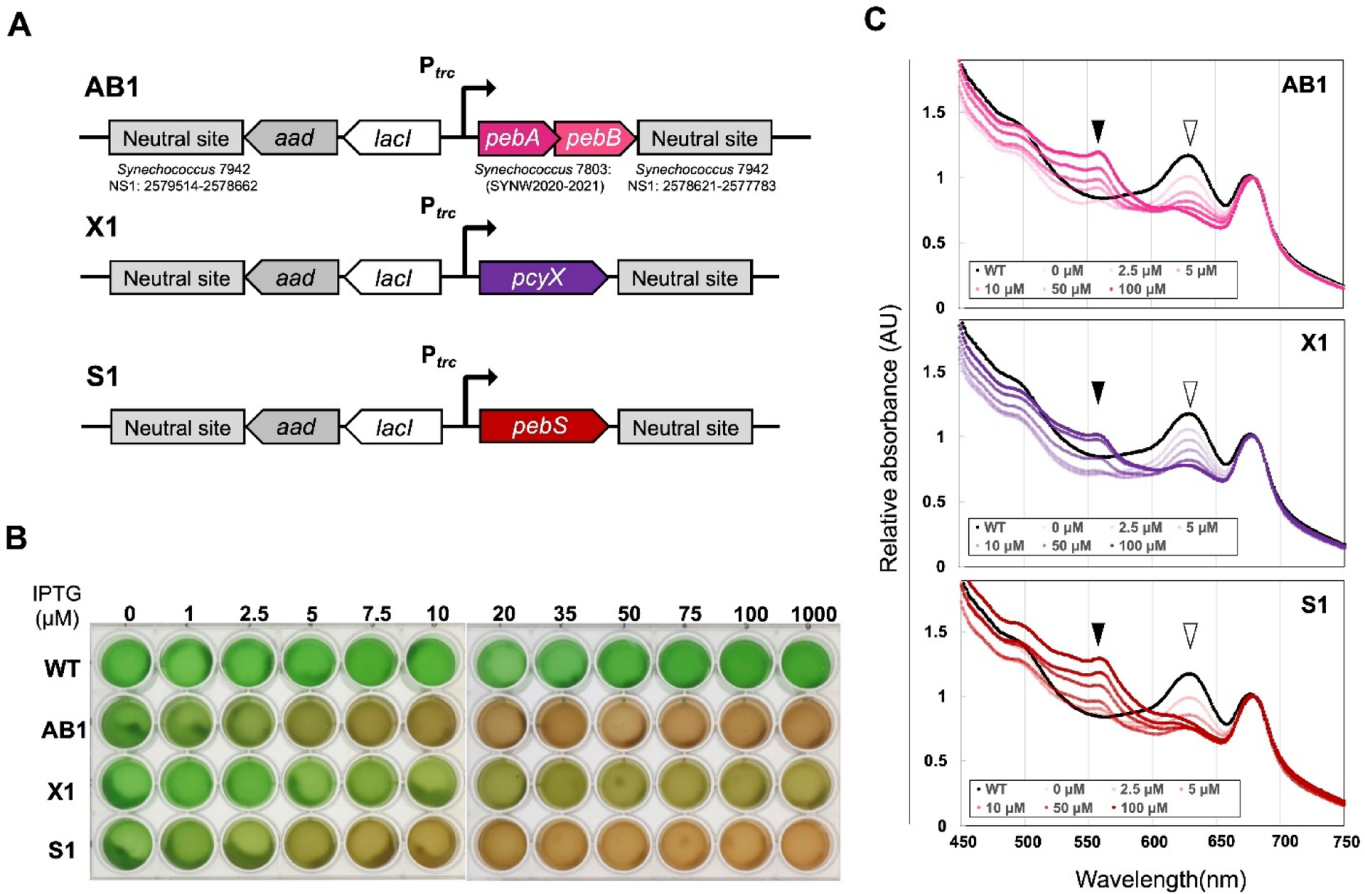
Construction of FDBR-expressing strains allowing control of PEB levels. (**A**) Schematic diagram of the genomic construct of *Synechococcus* 7942 strains expressing FDBRs (*pebA*-*pebB*, *pcyX*, and *pebS*) in an IPTG-dependent manner. The genes encoding FDBR were placed under the IPTG-dependent *trc* promoter and introduced into the neutral site 1 (NS1) of *Synechococcus* 7942 along with the spectinomycin resistance gene (*aad*) and *lacI*. (**B**) Effect of PEB synthase expression. After culturing PEB synthase-expressing and wild-type strains for one week, the strains were diluted to OD_750_ = 0.5 and further cultivated under white light (60 µmol photons m^−2^ s^−1^) with and without IPTG (**C**) Absorption spectrum of the cultures. The spectra were normalized at 680 nm. Closed arrowhead: peak at 560 nm and open arrowhead: peak at 625 nm. AB1, *pebA* and *pebB* co-expressing strain; X1, *pcyX* expressing strain; S1, *pebS* expressing strain; WT, wild type; IPTG, isopropyl ß-D-1-thiogalactopyranoside.

Three distinct enzymatic systems for PEB biosynthesis have been identified to date, each reported to exhibit distinct activities in *E. coli* cells^6,18,19^. To compare their activities directly in cyanobacteria, we constructed a series of *Synechococcus* 7942 strains expressing different FDBRs. The *pebA–pebB* operon, *pcyX*, and *pebS* were placed under the IPTG-inducible *trc* promoter and integrated into a neutral site (NS) on the *Synechococcus* 7942 chromosome. The resulting strains were designated *Synechococcus* 7942 AB1, X1, and S1, respectively (Fig. 2A).

Following IPTG induction, all three strains exhibited a brownish coloration in a concentration-dependent manner (Fig. 2B), although the extent of this change differed among strains. In particular, the X1 strain showed visibly weaker coloration compared with AB1 and S1 under the same conditions. Absorption spectra revealed a decrease in the 625-nm peak associated with phycocyanin (PC) and an increase in the 560-nm peak associated with phycoerythrobilin (PEB), both of which varied with IPTG concentration (Fig. 2C). Based on absorbance at 560 nm, the apparent activity of PcyX was lower than that of PebA–PebB and PebS, and was estimated to be less than one-tenth under the tested culture conditions.

### Spectral characteristics of cultures and PBS complexes in FDBR-expressing strains under low PEB induction

**Fig. 3:**
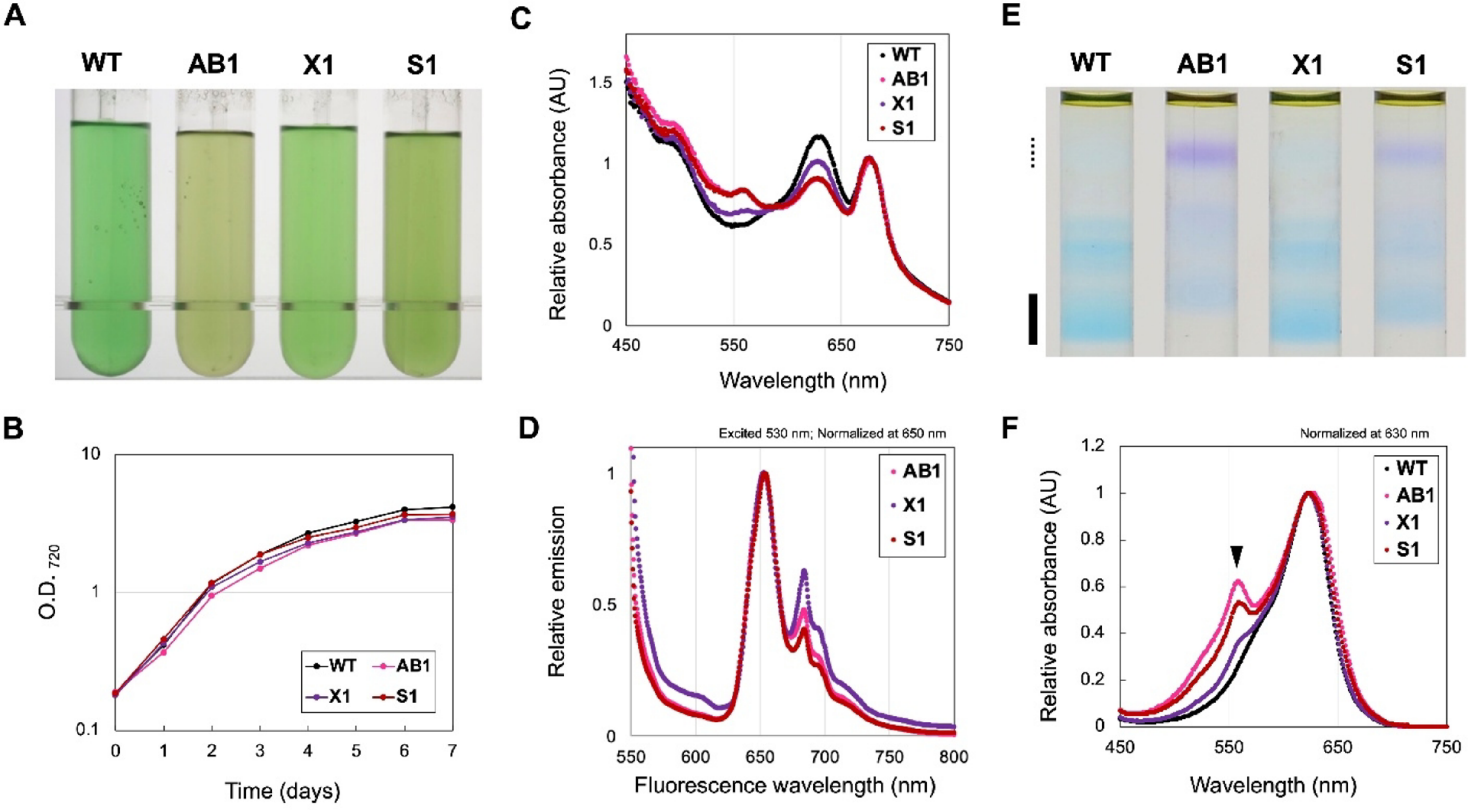
Spectral characteristics of culture and phycobilisome (PBS) complexes in FDBR-expressing strains. The spectral characteristics of cultures and PBS complexes in FDBR-expressing strains were compared. FDBR-expressing strains (AB1, X1 and S1) were cultured in BG-11 medium containing 5 µM IPTG with CO_2_ bubbling, whereas the wild-type strain (WT) was cultured without IPTG. (**A**) Images of culture one day after inoculation. (**B**) Growth curves under white light. (**C**) Absorption spectra of the cultures one day after inoculation. Spectra were normalized at 680 nm. (**D**) Low-temperature (77 K) fluorescence emission spectra excited at 530 nm. Spectra were normalized to the fluorescence intensity at 650 nm (phycocyanin emission). (**E**) Images of PBS fraction after SDG centrifugation. (**F**) Absorption spectra of the PBS complex fractions. Spectra of the bottom fraction containing mature PBS (bold line in **E**) normalized at 630 nm. Closed arrowhead indicate the peak at 560 nm.

In a previous study, we demonstrated that the accumulation of small amounts of PEB in PBS complexes altered the PBS properties of *Synechococcus* 7942 toward a green-light–adapted type^13^. To examine whether similar changes occur in the newly constructed strains, each strain was cultured with 5 µM IPTG, and the spectral characteristics of whole cells and PBS complexes were analyzed (Fig. 3).

Absorption spectra of whole cells under CO₂ bubbling conditions revealed that AB1 and S1 accumulated detectable levels of PEB-bound phycobiliproteins even at low IPTG induction, whereas X1 exhibited comparatively lower PEB accumulation (Fig. 3C). To evaluate energy transfer from green light (530 nm) among the three FDBR-expressing strains, low-temperature (77 K) fluorescence emission spectra were measured (Fig. 3D). Upon excitation at 530 nm, X1 displayed relatively higher fluorescence intensities corresponding to APC (685 nm), PSII (695 nm), and PSI (720 nm) compared with AB1 and S1. These observations suggest that even low-level PEB incorporation can modify PBS spectral properties while maintaining functional coupling to the photosystems.

Cell extracts from the wild type (WT) and the three FDBR-expressing strains were subjected to sucrose density gradient (SDG) centrifugation to separate intact PBS complexes from their components. In the AB1 and S1 strains, the fraction corresponding to full-size PBS complexes, typically detected at the bottom of the gradient, shifted toward an upper position (Fig. 3E, solid line), indicating a reduction in complex size. In addition, red-colored fractions appeared in the upper region of the gradient (Fig. 3E, dashed line), consistent with disassembled phycobiliproteins reported previously^13^. In contrast, X1 showed a distribution pattern more similar to WT. No notable differences in growth were observed under white-light conditions (Fig. 3B), suggesting that the reduction in PBS complex size does not substantially affect cell proliferation under these culture conditions.

The absorption spectra of PBS fractions from the FDBR-expressing strains (Fig. 3E, solid line) exhibited an additional peak near 560 nm, corresponding to PE, in addition to the 620-nm peak associated with PC. The 560-nm peak was most pronounced in AB1, followed by S1 and X1, consistent with the whole-cell absorption spectra (Fig. 3C). These results indicate that PEB accumulates within the PBS complexes under low-level FDBR induction and that partial degradation and size reduction of the PBS complexes occur in the AB1 and S1. The relative intensity of the 560-nm peak further suggest that the apparent activities of the introduced FDBRs in *Synechococcus* 7942 follow the order PebA–PebB > PebS > PcyX.

### Effect of PEB binding on the PBS complex

To investigate the basis of the size reduction in PEB-containing PBS complexes, proteins from WT and AB1 PBS fractions were fluorescently labeled and analyzed by SDS–PAGE (Supplementary Fig. S1A). Comparison of the fluorescence intensities of rod–rod and rod–core linker proteins showed a reduction in the 30-kDa rod–rod linker protein relative to other linker proteins in AB1 (Fig. S1B). As the 30-kDa rod–rod linker has been reported to occupy the outermost position in the PBS rod and to be among the first components affected by changes in light conditions^20,21^, these results suggest that PEB accumulation may promote dissociation of the outer rod disc, contributing to PBS size reduction.

**Fig. 4:**
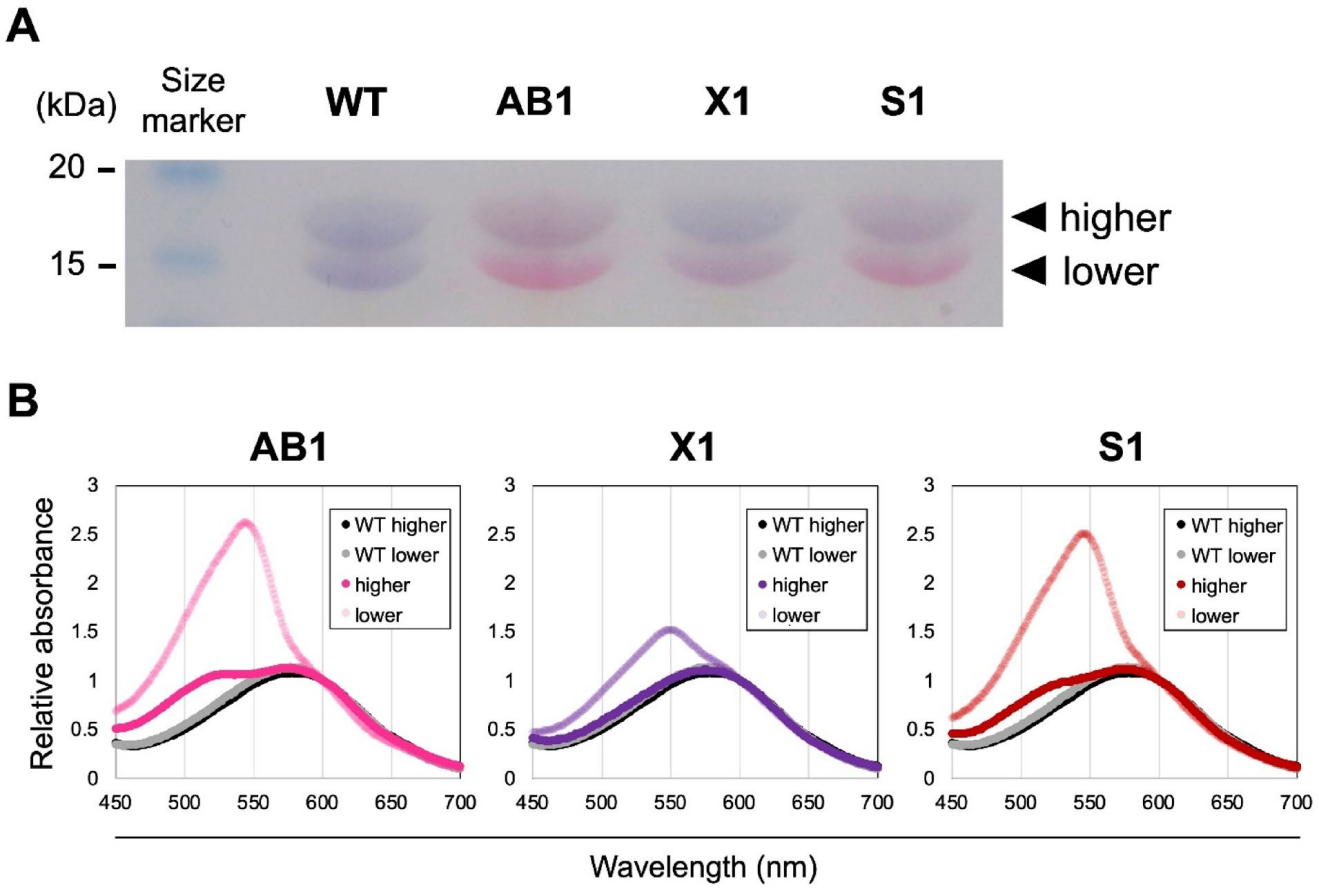
Binding of PEB to apoproteins. The cultures were grown under white light with 5 µM IPTG and used for the analysis. (**A**) Cropped coomassie brilliant blue-stained gel images of phycobiliproteins. The complete image data for the gel is shown in the Additional Information. The arrows indicate the lower- and higher-molecular-weight bands of phycobiliproteins. Based on their migration positions, these bands are consistent with disc proteins (e.g., CpcA and CpcB), although overlap with core proteins cannot be excluded. Therefore, the fractions are referred to here simply as the “lower-” and “higher-” molecular-weight bands. (**B**) Absorption spectra of phycobiliproteins extracted gel shown in (**A**). Each set of spectra was normalized to 584 nm.

Since PEB has been reported to bind to apoproteins of PCB-type PBSs^11–13,22^, we next examined phycobiliproteins under conditions of PEB accumulation. Protein extracts were prepared from cells cultured with 5 µM IPTG for FDBR induction and analyzed by SDS–PAGE. Two colored bands were observed (Fig. 4A), hereafter referred to as the lower- and higher-molecular-weight bands. The migration positions of these bands are consistent with those expected for disc proteins (CpcA/CpcB), although they also overlap with phycobilisome core proteins such as ApcA and ApcB. Given the relative abundance of rod (disc) proteins compared to core components in *Synechococcus* 7942, the major constituents of these bands are likely disc proteins; however, minor contributions from core proteins cannot be excluded.

In the AB1 and S1 strains, both the higher- and lower-molecular-weight bands exhibited red coloration, whereas in the X1 strain, red coloration was predominantly detected in the lower-molecular-weight band. Proteins from each band were extracted, and their absorption spectra were compared with those of the WT. In AB1 and S1, extracts from both bands showed a shift toward shorter wavelengths, while in X1, a shift was observed only for the lower-band extract (Fig. 4B). Notably, the lower-molecular-weight bands appeared visually redder than the corresponding higher-molecular-weight bands, and the absorption peak of the lower-band extracts was markedly higher in all three FDBR-expressing strains. Taken together, these results indicate that PEB incorporation is preferentially enhanced in the lower-molecular-weight band. Based on expected migration behavior and spectral characteristics, this band most likely corresponds to CpcA, consistent with previous reports^11,13^.

### Growth advantage of FDBR-expressing strain under green light

**Fig. 5:**
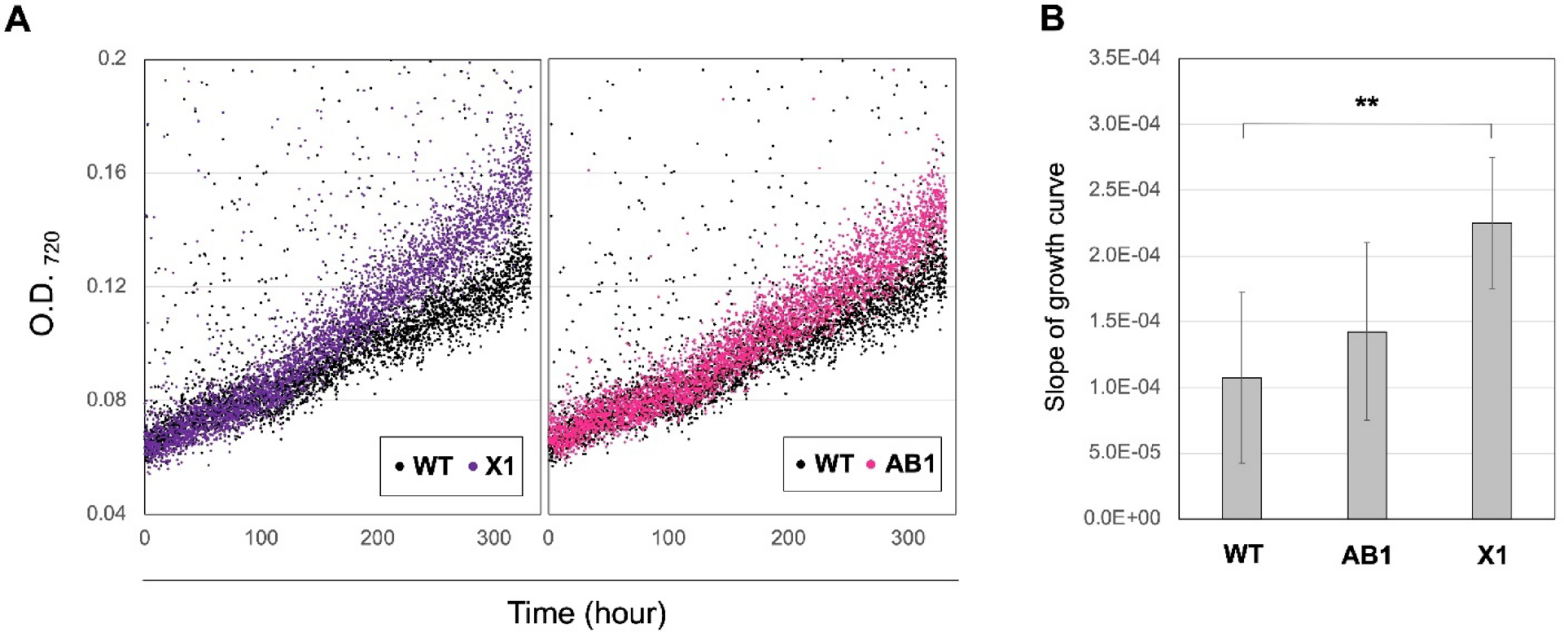
Promotion of growth under green light conditions through FDBR expression. (**A**) Growth curves of WT, X1, and AB1 cultures. (**B**) Comparison of growth rate estimated from the slopes of the growth curves. Statistical significance was evaluated using a paired *t*-test in Microsoft Excel (**p < 0.05; WT vs. X1).

Because the PBSs of the FDBR-expressing strains accumulated PEB and were able to absorb green light, we next evaluated whether this conferred a growth advantage under green-light illumination. The growth of the AB1 and X1 strains was compared with that of the WT. Under white-light conditions, no notable differences were observed among the strains (Fig. 3B). In contrast, under green-light conditions, both AB1 and X1 exhibited faster growth than the WT (Fig. 5A). Analysis of growth rates based on the slopes of the growth curves indicated that X1 showed a significantly higher growth rate than WT (Fig. 5B). These results indicate that PEB accumulation is associated with enhanced proliferation under green light, and further suggest that proliferation is promoted when the PBS complex is maintained in a nearly complete state, consistent with our previous observations^13^.

### Effects of PEB accumulation on gene expression

**Fig. 6:**
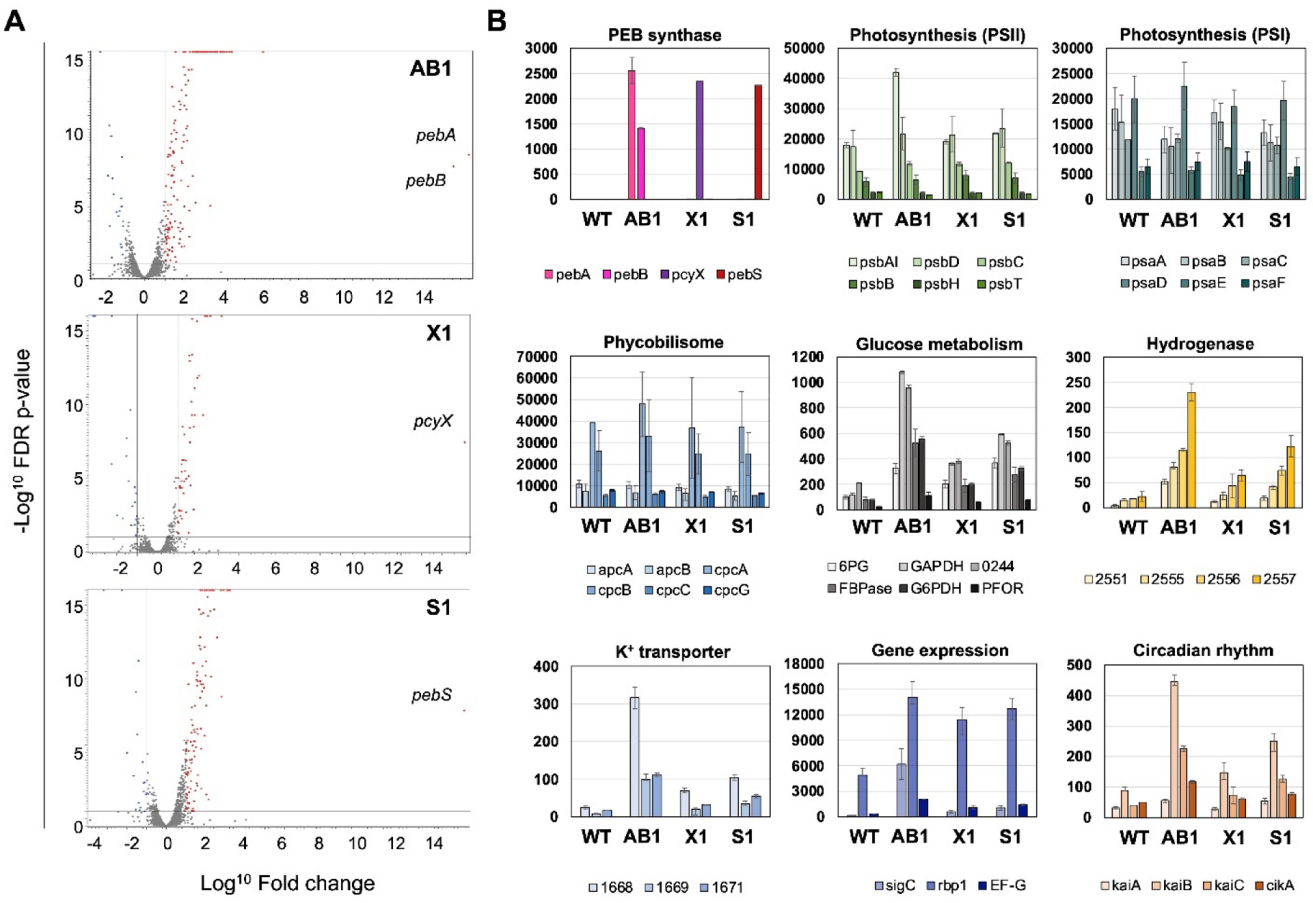
Comparative transcriptomic analysis of *Synechococcus* 7942 expressing different FDBRs. Results of the comparison between WT and FDBR-expressing strains (AB1, X1, and S1). After 4 days cultivation under white light with 5 µM IPTG (for PEB expressing strain) and CO_2_ bubbling, the cells were harvested and subjected for the RNAseq analysis. (**A**) Volcano plot of significantly deferentially expressed genes. Upregulated and downregulated genes are depicted in red and blue, respectively (log_2_ FC ≥ │1│, FDR *p* ≤ 0.1). The X-axis corresponds to log_2_ fold change, and the Y-axis displays −log^10^ of an adjusted *p* value of FDR. (**B**) The expression levels of genes (TPM values) showing a significantly increased expression compared to the wild-type strain. Data were extracted from Supplementary Table S1 and presented in the graph.

Because bilins function not only in light harvesting but also in the regulation of gene expression through binding to CBCR photoreceptors, changes in bilin metabolism are expected to elicit broad transcriptional responses. To evaluate the effects of PEB accumulation on gene expression in *Synechococcus* 7942, we performed RNA-seq analysis using the WT strain and three FDBR-expressing strains. Total RNA was extracted from cultures grown under white light for 4 days in the presence of 5 µM IPTG (for strains AB1, S1, and X1) and subjected to sequencing analysis. The TPM (transcripts per million) values, fold ratios, and FDR-adjusted *p* values for the WT and each FDBR-expressing strain are summarized in Supplementary Table S1A, and the resulting volcano plots are shown in Fig. 6A (without ORF IDs) and Supplementary Fig. S2 (with ORF IDs).

Among the three FDBR-expressing strains, the expression levels of the heterologously introduced *pebA*–*pebB*, *pcyX*, and *pebS* genes in *Synechococcus* 7942 were comparable (Fig. 6B), indicating that the observed differences among strains are attributable to enzymatic activity rather than expression level. Relative to the WT strain, no significant changes were detected in the expression of genes encoding components of PSI, PSII, or the phycobilisome. However, 39 genes were commonly upregulated and 6 were commonly downregulated across all three FDBR-expressing strains (Supplementary Fig. S3 and Supplementary Table S1B). The commonly downregulated genes did not clearly cluster into a specific functional category, whereas the commonly upregulated genes included those associated with glucose metabolism, hydrogenase activity, potassium transport, and regulatory factors, such as sigma factors and circadian clock–related genes (Fig. 6B). Importantly, the magnitude of these transcriptional changes was highest in AB1, moderate in S1, and lowest in X1, demonstrating that the extent of gene expression remodeling positively correlates with PEB accumulation levels (Figs. 2C and 3C). These findings indicate that increased PEB levels exert a stronger influence on transcriptional responses, leading to broader remodeling of cellular metabolism.

We observed a coordinated upregulation of genes involved in glucose metabolism—including 6-phosphogluconate dehydrogenase (6PG; Synpcc7942_0039), glyceraldehyde-3-phosphate dehydrogenase (GAPDH; Synpcc7942_0245), D-fructose 1,6-bisphosphatase (FBPase; Synpcc7942_2335), glucose-6-phosphate dehydrogenase (G6PDH; Synpcc7942_2334), pyruvate: ferredoxin (flavodoxin) oxidoreductase (PFOR; Synpcc7942_2384), and glycogen/α-glucan phosphorylase (Synpcc7942_0244)—together with genes encoding the NAD-reducing hydrogenase HoxS subunits (Synpcc7942_2551, 2555–2557). Central carbon metabolism in cyanobacteria directly regulates NAD(P)H turnover; thus, modulation of these pathways influences cellular redox balance by redistributing excess reducing equivalents. The upregulation of these genes therefore likely reflects a cellular adjustment to redox imbalance under conditions of PEB accumulation^23,24^. In addition, the induction of genes encoding a K^+^ transport system (Synpcc7942_1668–1671) in the FDBR-expressing strains suggests that PEB accumulation is accompanied by transcriptional changes in ion transport pathways, potentially reflecting a response related to osmotic or ionic homeostasis^25^.

We also detected increased expression of several genes associated with transcriptional, post-transcriptional, and translational regulation in response to PEB accumulation. The group 2 sigma factor SigC (Synpcc7942_1849) and the RNA-binding protein Rbp1 (Synpcc7942_1999), both known to respond to darkness and cold stress^26–28^, showed elevated transcript levels. In addition, the genes encoding the circadian clock components KaiB (Synpcc7942_1217) and KaiC (Synpcc7942_1216) were upregulated in AB1 and S1, the strains with higher PEB levels, consistent with their roles in broad transcriptional regulation^29^. The gene encoding the translation elongation factor EF-G (Synpcc7942_2082) was also increased, aligning with previous reports that translation elongation factors contribute to PSII repair under oxidative stress conditions^30^.

Finally, we note that the expression levels of the *nblA* gene were significantly increased in the AB1 and S1 strains (Table S1, Supplementary Fig. S4), which accumulate relatively large amounts of PEB. Compared with the WT, 3.27- and 2.66-fold increases in *nblA* expression were observed in AB1 and S1, respectively. Because NblA plays a central role in PBS degradation and remodeling under several stresses^31–33^, this differential expression may reflect distinct regulatory or structural adjustments to altered phycobiliprotein composition in the engineered strains. In particular, the increased *nblA* expression in AB1 and S1 could be associated with reduced stability or modified assembly of CpcA-containing hexamers incorporating PEB, consistent with the differences observed in Fig. 3. Overall, these observations indicate that PEB accumulation is accompanied by broader changes in regulatory gene expression, potentially influencing cellular physiology through multiple regulatory layers.

## Discussion

In summary, our results clarify the relative behavior of the three FDBRs within cyanobacterial cells and their impact on PBS function. When expressed under identical conditions, their apparent activities followed the order PebA–PebB > PebS > PcyX. Given that PebA–PebB are broadly conserved among cyanobacteria^10^, this likely reflects a higher degree of compatibility with the native cellular environment compared with PebS or PcyX (Figs. 2 and 3). By contrast, PcyX—originating from metagenome-derived sequences^6,9^—exhibited lower apparent activity, which may allow more gradual modulation of intracellular PEB levels. Importantly, our findings demonstrate that effective adaptation of PBSs toward green-light utilization in *Synechococcus* 7942 requires PEB accumulation at levels that preserve PBS structural integrity. Despite a lower overall level of PEB accumulation, the PcyX-expressing strain (X1) showed enhanced energy transfer from phycobiliproteins to PSII and PSI upon 530 nm excitation relative to AB1 and S1 (Fig. 3D), suggesting that its PBS configuration is particularly well suited for green-light energy capture and transfer. These results indicate that controlled PEB accumulation—rather than maximal production—may favor the functional incorporation of PEB into intact PBS complexes. Furthermore, the observation that PebS alone supported PEB accumulation comparable to that of PebA–PebB highlights that both PebS and PcyX represent practical tools for tuning PBS spectral properties. Together, these FDBRs provide complementary strategies for engineering cyanobacterial light-harvesting systems for future synthetic biology applications.

This study provides new insight into the interaction between PEB and PBS complex in *Synechococcus* 7942. Consistent with previous reports^11–13^, our results show that PEB incorporation is preferentially enhanced in the lower-molecular-weight phycobiliprotein band, consistent with CpcA based on electrophoretic mobility and spectral characteristics. These findings suggest that CpcA has a greater propensity to associate with PEB than other phycobiliprotein subunits in cyanobacterial cells. Under conditions inducing modest PEB accumulation in the AB1 and S1 strains (5 µM IPTG), red coloration and spectral shifts were detected in both the lower- and higher-molecular-weight bands, indicating that additional phycobiliprotein components are capable of incorporating PEB once intracellular levels exceed a certain threshold. Notably, *Synechococcus* 7942 possesses a relatively rod-rich phycobilisome architecture compared with other model cyanobacteria, such as *Synechococcus* sp. PCC 7002 and *Synechocystis* sp. PCC 6803. This structural characteristic supports the interpretation that the higher-molecular-weight band predominantly represents rod proteins, including CpcB. These conditions were also accompanied by a reduction in PBS complex size, consistent with dissociation of the outermost rod disc. Although the mechanistic sequence remains unclear, these observations suggest that altered bilin occupancy may influence PBS stability and organization. A detailed identification of the specific chromophore-binding sites and their occupancy would further clarify the mechanism of PEB incorporation; however, such analyses require structural or proteomic approaches and remain an important direction for future work. Collectively, these findings refine our understanding of PBS assembly by highlighting the interplay between bilin loading, phycobiliprotein, and linker-dependent structural maintenance.

Furthermore, the intracellular state during PEB accumulation was investigated through transcriptome analysis. Although FDBRs consume electrons from reduced ferredoxin, expression of heterologous FDBRs unexpectedly resulted in transcriptomic signatures suggestive of a shift toward a more reduced intracellular redox environment, likely due to altered electron distribution. Significant changes in gene expression were observed, including upregulation of genes involved in glucose metabolism, HoxS hydrogenase components, and potassium transporters, correlating with PEB accumulation levels. Additionally, fluctuations were noted in the expression of proteins related to gene expression regulation, such as SigC, Rbp1, EF-G, and KaiBC. These results suggest that these genes may contribute to mitigating the altered intracellular redox balance induced by PEB accumulation. Notably, *nblA* expression was significantly increased in the AB1 and S1 strains, which accumulate relatively high levels of PEB (Table S1, Supplementary Fig. S4). Because NblA functions as a key adaptor protein mediating phycobilisome degradation under several stress conditions^31–33^, its upregulation suggests that PEB accumulation may influence PBS stability or remodeling. One possible interpretation is that incorporation of PEB into CpcA-containing hexamers alters phycobilisome structural integrity, thereby triggering a partial degradation or remodeling response. Whether NblA directly contributes to the apparent destabilization of PBS complexes in the FDBR-expressing strains remains unclear and warrants further investigation. Ongoing genetic analyses of *nblA* in the FDBR-expressing background will help clarify its role in PBS remodeling under PEB-accumulating conditions.

While these observations are consistent with redox-associated regulatory responses, they do not exclude the possibility that bilin accumulation itself may act as a signaling cue. Bilins are known to function not only as light-harvesting pigments but also as chromophores in photoreceptors. Thus, beyond redox homeostasis, altered intracellular bilin composition may influence regulatory pathways through pigment-binding photoreceptors. One possibility is the modulation of gene expression through binding to the GAF domain of CBCR-type photoreceptors^14,15^. In *Synechococcus* 7942, two proteins with pigment-binding GAF domains have been identified: the PixJ homologue (Synpcc7942_0858) and a protein (Synpcc7942_2534) containing both a GAF domain and a GGDEF cyclic-di-GMP synthesis motif. In the closely related species *Synechococcus* 3055, PixJ functions in phototaxis^17^; however, *Synechococcus* 7942 does not exhibit phototaxis, and no significant changes in phototaxis-related gene expression were observed even under conditions of PEB accumulation. This suggests that bilin-mediated signaling in *Synechococcus* 7942 may operate through regulatory mechanisms distinct from phototaxis, potentially linking bilin accumulation to broader cellular responses rather than behavioral outputs.

Together, these findings point to a previously unrecognized connection between bilin biosynthesis, redox balance, and cellular signaling. Importantly, our results demonstrate that targeted manipulation of pigment biosynthesis can trigger broad metabolic and regulatory reorganization, underscoring the necessity of system-level analyses when engineering photosynthetic organisms. Disentangling both the metabolic and regulatory consequences of PEB accumulation will therefore be essential for developing predictive and robust synthetic biology strategies aimed at redesigning light-harvesting systems.

## Methods

Except for the transcriptome analysis described below, all other experiments were performed largely as described in our previous study^13^, with minor modifications.

### Culture conditions for cyanobacteria

The cyanobacterium *Synechococcus* 7942 WT strain and its derivatives were grown photoautotrophically at 30 °C under continuous WL illumination (60 µmol photons m^−2^ s^−1^). Cells were cultured in a modified BG-11 medium containing double the usual amount of sodium nitrate (final concentration = 35.3 mM) and 20 mM 4-(2-hydroxyethyl)-1-piperazineethanesulfonic acid (HEPES)–KOH (pH 8.2) with continuous bubbling of 2% CO_2_. When required, spectinomycinwas added to the medium at a final concentration of 40 µg mL^−1^.

Growth experiments under GL conditions were performed using a Multi-Cultivator MC1000-OD MIX (Photon Systems Instruments, Czech Republic) with continuous GL illuminations (530 nm; 60 µmol photons m^−2^ s^−1^) and bubbling of ambient air. After pre-incubation on a BG-11 agar plates for 1 week, cells were harvested and inoculated into liquid BG-11 medium containing IPTG at an initial OD_720_ of 0.05, followed by cultivation for 1 week. Growth was monitored by measuring OD_720_ over 7 days (168 h) and approximate growth curves were constructed from these values. All measurements were performed in triplicate, and growth rates were compared based on the slope of the growth curves. Statistical significance was evaluated using a paired *t*-test in Microsoft Excel.

### Strain construction

DNA fragments were amplified using KOD One DNA polymerase (TOYOBO, Japan) and subcloned into the vector plasmids using the In-Fusion HD Cloning Kit (TaKaRa, Japan). Codon-optimized FDBR genes, *pcyX* and *pebS*, which were designed and synthesized by Eurofins Genomics (Ebersberg, Germany), were amplified using specific primer sets (F1/R2 for *pcyX* and F3/R4 for *pebS*, Supplementary Tables S2 and S3). The amplified fragments were cloned into the pBNS vector, which had been amplified using the primer set (F5/R6), generating the plasmids pBNS-pcyX and pBNS-pebS, respectively. To examine the effects of PEB synthesis using an identical expression construct, the *pebA*-*pebB* operon in *Synechococcus* sp. PCC 7803 was amplified using primer set F7 and R8 and cloned into the same vector backbone to generate pBNS-pebAB. These plasmids were introduced into *Synechococcus* 7942, and spectinomycin-resistant transformants were obtained. Successful transformation of strains with plasmids was confirmed by PCR amplification using specific primer sets (F9/R10) and the resulting strain was named *Synechococcus* 7942 X1, S1, and AB1, respectively.

### Analysis of phycobiliproteins

Cells were harvested and resuspended in 100 µL of A buffer (10% glycerol, 100 mM NaCl, 20 mM HEPES–NaOH; pH 7.5) and then disrupted with glass beads using a bead beater (Micro Smash MS-100R, TOMY Seiko Co., Tokyo, Japan). After the centrifugation at 20,000 × *g* for 1 min at 18 °C, 60 µL of the supernatant was transferred to a fresh tube and mixed with 240 µL of acetone (final concentration = 80%) to remove chlorophyll and carotenoids from the cell lysates. The mixture was centrifuged again at 20,000 × *g* for 1 min, yielding pellets enriched in phycobiliproteins. Bilins binding to apoproteins was analyzed by SDS-PAGE. The phycobilin pellets were resuspended and 20 µL of each sample was mixed with loading buffer (final concentration = 0.0625 Tris-HCl, 10% glycerol, 2% SDS, and 0.01% bromophenol blue) and separated on a 15% (w/v) polyacrylamide gel. After electrophoresis, gel bands were excised and extracted using ATTOPREP MF (ATTO, Tokyo, Japan), and the absorption spectra of the extracted phycobiliproteins were measured.

### Isolation of PBSs by SDG centrifugation

*Synechococcus* 7942 cultures (OD_750_ = 1.0; 30 mL) were harvested by centrifugation at 3,000 × *g* for 10 min at 25 °C. The cell pellets were washed once with 1 mL of 0.6 M potassium phosphate (KP) buffer (pH 7.0), centrifuged again under the same conditions, and stored at − 80 °C until analysis. For PBS isolation, the cells were thawed, washed twice with 0.6 M KP buffer, and resuspended in 0.6 mL of the same buffer. Cells were disrupted by vortexing with glass beads, and PBS complexes were extracted from thylakoid membranes by incubation with Triton X-100 (final concentration = 2%) for 15 min with gentle voltexing. After centrifugation at 20,000 × *g* for 20 min at 18 °C, 200 µL of the supernatant was loaded onto linear sucrose density gradients (10–50% sucrose in 0.6 M KP buffer) prepared in 14 × 89 mm open-top thinwall ultraclear tubes (Beckman Coulter, CA, USA) using a Gradient Master. The gradients were centrifuged at 154,300 × *g* for 18 h at 18 °C using SW41Ti rotor in an Optima XE-90 ultracentrifuge (Beckman Coulter).

### Spectrometry

Absorption spectra of whole cells, isolated phycobiliproteins, and PBS complexes were measured at 25 °C using a spectrophotometer (UV-1800, Shimadzu, Japan) or a multimode plate reader (VICTOR Nivo, PerkinElmer). Fluorescence excitation spectra were measured with emission monitored at 685 nm to detect APC fluorescence using a spectrophotometer (FP-8200, JASCO, Japan).

### Transcriptome analysis

Cells were inoculated into liquid BG-11 medium at an initial optical density (O.D. _750_) of 0.05, supplemented with 5 µM IPTG, and cultivated for 4 days under continuous illumination at 60 µmol photons m^−2^s^−1^ with 2% CO_2_ bubbling. Total RNA was extracted from exponentially growing cultures of *Synechococcus* 7942 wild-type and engineered strains expressing *pebA*–*pebB* (AB1), *pcyX* (X1), or *pebS* (S1) as described previously^34^. Ribosomal RNA was removed using NEBNext rRNA Depletion Kit (Bacteria) (New England Biolabs). Sequencing libraries with an average insert size of approximately 200 bp were prepared according to the manufacturer’s instructions using the NEBNext Ultra II Directional RNA Library Prep Kit for Illumina (New England Biolabs). A total of 12 libraries (three biological replicates per strains) were sequenced on the Illumina NextSeq 1000 platform. Raw reads were trimmed and quality-filtered using CLC Genomics Workbench ver. 25.0.2 (QIAGEN, Hilden, Germany). Trimmed reads were mapped to the all genes in *Synechococcus* 7942 (accession number: CP000100, CP000101) as well as to plasmids carrying FDBR (pBNS_pebAB, pBNS_pebS, and pBNS_pcyX, Supporting information). Gene expression levels were normalized and quantified as transcripts per million (TPM). Differentially expressed genes (DEGs) were identified by pairwise comparisons between WT and each engineered strain (WT vs. AB1, WT vs. X1, and WT vs. S1). Venn diagrams were generated to visualize the overlap of DEGs among comparisons, and volcano plots were used to display the magnitude and statistical significance of expression changes. TPM values of selected genes were plotted to compare expression patterns among the strains. The sequencing data underlying this study are available in the DDBJ Sequence Read Archive (DRA/SRA) under accession numbers DRR803588–DRR803599, within BioProject PRJDB39746.

## Supplementary Information

Below is the link to the electronic supplementary material.

Supplementary Material 1

## Data Availability

The sequencing data underlying this study are available in the DDBJ Sequence Read Archive (DRA/SRA) under accession numbers DRR803588–DRR803599, within BioProject PRJDB39746.

## Abbreviations

PBS: Phycobilisome
PC: Phycocyanin
PE: Phycoerythrin
PEC: Phycoerythrocyanin
APC: Allophycocyanin
FDBR: Ferredoxin-dependent bilin reductase
PCB: Phycocyanobilin
PEB: Phycoerythrobilin
PSII: Photosystem II
PSI: Photosystem I
NS: Neutral site
IPTG: Isopropyl ß-D-1-thiogalactopyranoside
SDG: Sucrose density gradient
WL: White light
GL: Green light
