# Supplementary material for: Comparative study of phycoerythrobilin synthases for fine-tuning photosynthetic light-harvesting complexes, phycobilisomes
Source: Sci Rep. 2026 Apr 27;16:15992. doi: 10.1038/s41598-026-50582-3 (PMC13197396; doi:10.1038/s41598-026-50582-3)

Additional Information

Comparative Study of Phycoerythrobilin Synthases for Fine-Tuning Photosynthetic Light-Harvesting Complexes, Phycobilisomes

Mizuho Sato, Mai Watanabe, Misaki Iwata, Kaisei Maeda, Kaori Nimura-Matsune, Masahiko Ikeuchi, Rei Narikawa, and Satoru Watanabe*

*** Correspondence:** Corresponding Author: s3watana@nodai.ac.jp

1. Sequence files of pBNS-pcyX, -pebS, and -pebAB

2. Figures

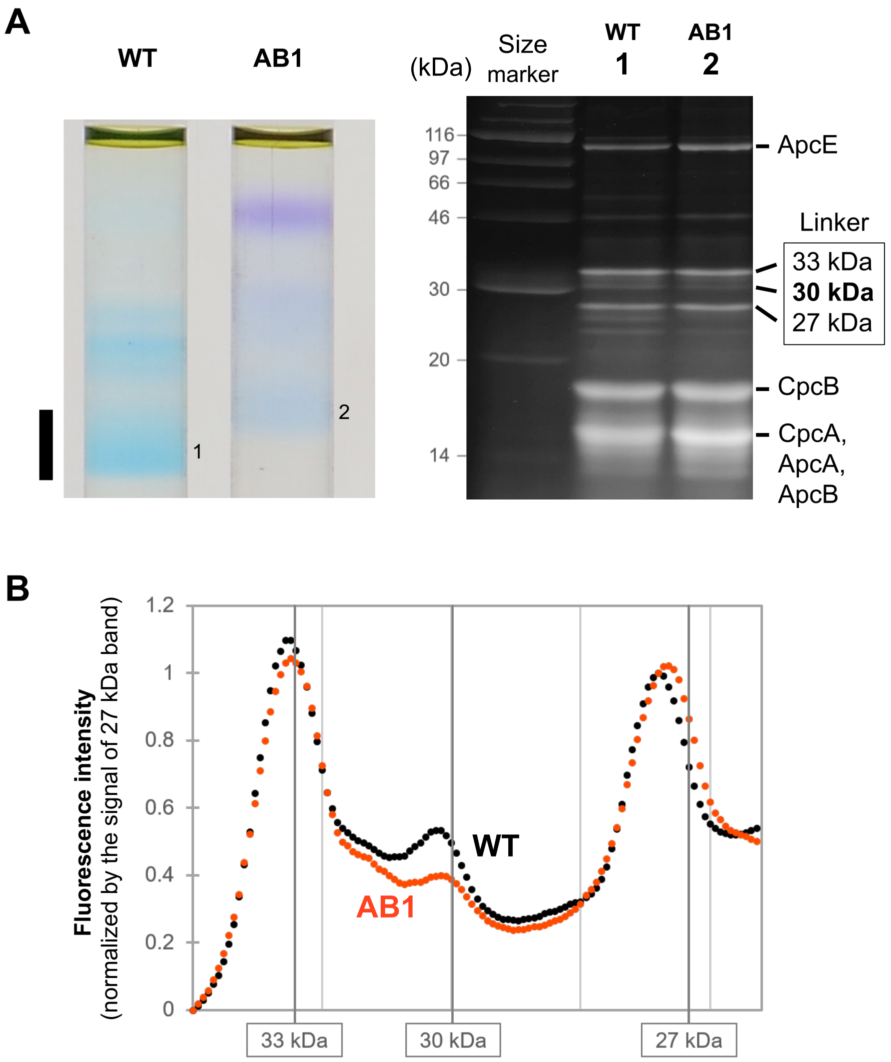

Supplementary Figure S1. Comparison of the linker protein content in PBS complexes

Phycobilisome complexes from wild-type (WT) and AB1, fractionated by SDG, were fluorescently labelled and separated by SDS-PAGE to compare the quantities of linker proteins (33, 30, and 27 kDa). (A) PBS fraction image after SDG centrifugation (part of Fig. 3A) and SDS-PAGE image of PBS complex proteins stained with fluorescent reagent (EzLabel FluoroNeo, ATTO Co., WSE-7010)). (B) Comparison of the fluorescence intensity of the linker protein around 30 kDa as shown in gel image A.

AB1

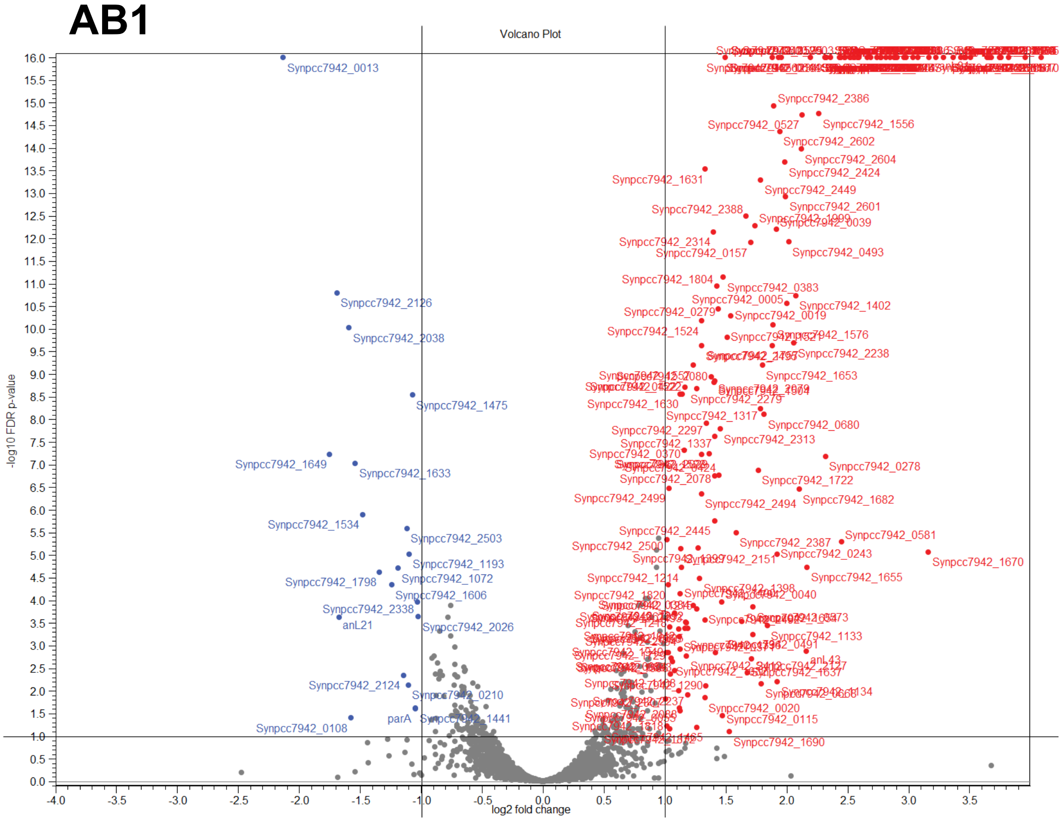
Volcano Plot
-log10 FDR p-value
log2 fold change
Synpcc7942_0013
Synpcc7942_2386
Synpcc7942_0527
Synpcc7942_1556
Synpcc7942_2602
Synpcc7942_2604
Synpcc7942_2424
Synpcc7942_1631
Synpcc7942_2449
Synpcc7942_2601
Synpcc7942_2388
Synpcc7942_0039
Synpcc7942_2314
Synpcc7942_0157
Synpcc7942_0493
Synpcc7942_1804
Synpcc7942_0383
Synpcc7942_0005
Synpcc7942_2126
Synpcc7942_1402
Synpcc7942_0279
Synpcc7942_0019
Synpcc7942_2038
Synpcc7942_1524
Synpcc7942_1576
Synpcc7942_2238
Synpcc7942_1653
Synpcc7942_1475
Synpcc7942_1630
Synpcc7942_1317
Synpcc7942_0680
Synpcc7942_2297
Synpcc7942_1337
Synpcc7942_2313
Synpcc7942_1649
Synpcc7942_0370
Synpcc7942_0278
Synpcc7942_1633
Synpcc7942_2078
Synpcc7942_1722
Synpcc7942_2499
Synpcc7942_2494
Synpcc7942_1682
Synpcc7942_1534
Synpcc7942_2445
Synpcc7942_2503
Synpcc7942_2387
Synpcc7942_0581
Synpcc7942_2500
Synpcc7942_0243
Synpcc7942_1670
Synpcc7942_1193
Synpcc7942_1798
Synpcc7942_1072
Synpcc7942_1214
Synpcc7942_1655
Synpcc7942_1606
Synpcc7942_1398
Synpcc7942_0040
Synpcc7942_2338
Synpcc7942_2026
anL21
Synpcc7942_1133
Synpcc7942_0491
anL43
Synpcc7942_1637
Synpcc7942_2124
Synpcc7942_0210
Synpcc7942_0020
parA
Synpcc7942_1441
Synpcc7942_0115
Synpcc7942_0108
Synpcc7942_1690

X1

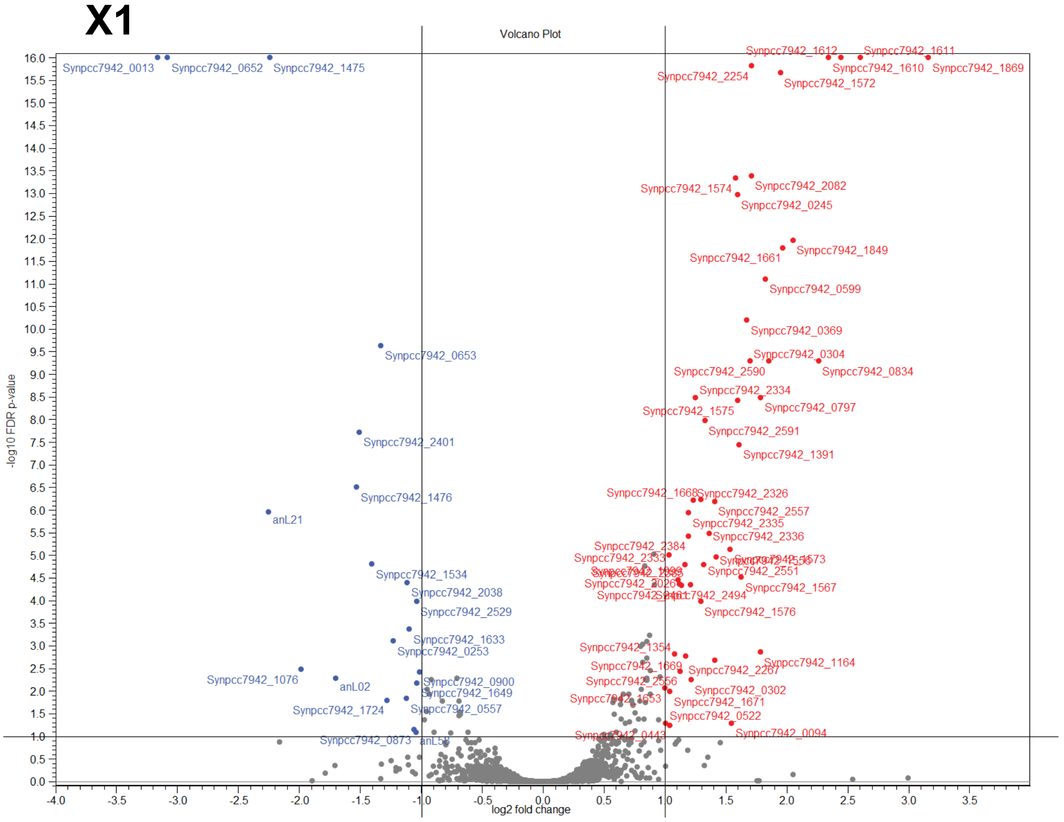
Volcano Plot
-log10 FDR p-value
log2 fold change
Synpcc7942_0013
Synpcc7942_0652
Synpcc7942_1475
Synpcc7942_1612
Synpcc7942_1611
Synpcc7942_2254
Synpcc7942_1610
Synpcc7942_1869
Synpcc7942_1572
Synpcc7942_1574
Synpcc7942_2082
Synpcc7942_0245
Synpcc7942_1849
Synpcc7942_1661
Synpcc7942_0599
Synpcc7942_0369
Synpcc7942_0653
Synpcc7942_0304
Synpcc7942_2590
Synpcc7942_0834
Synpcc7942_2334
Synpcc7942_1575
Synpcc7942_0797
Synpcc7942_2401
Synpcc7942_2591
Synpcc7942_1391
Synpcc7942_1476
Synpcc7942_1668
Synpcc7942_2326
anL21
Synpcc7942_2557
Synpcc7942_2335
Synpcc7942_2336
Synpcc7942_2384
Synpcc7942_2353
Synpcc7942_1534
Synpcc7942_2551
Synpcc7942_2038
Synpcc7942_1567
Synpcc7942_2494
Synpcc7942_2529
Synpcc7942_1576
Synpcc7942_1633
Synpcc7942_0253
Synpcc7942_1354
Synpcc7942_1164
Synpcc7942_1669
Synpcc7942_1076
anL02
Synpcc7942_0900
Synpcc7942_1649
Synpcc7942_0302
Synpcc7942_1671
Synpcc7942_1724
Synpcc7942_0557
Synpcc7942_0522
Synpcc7942_0094
Synpcc7942_0873

S1

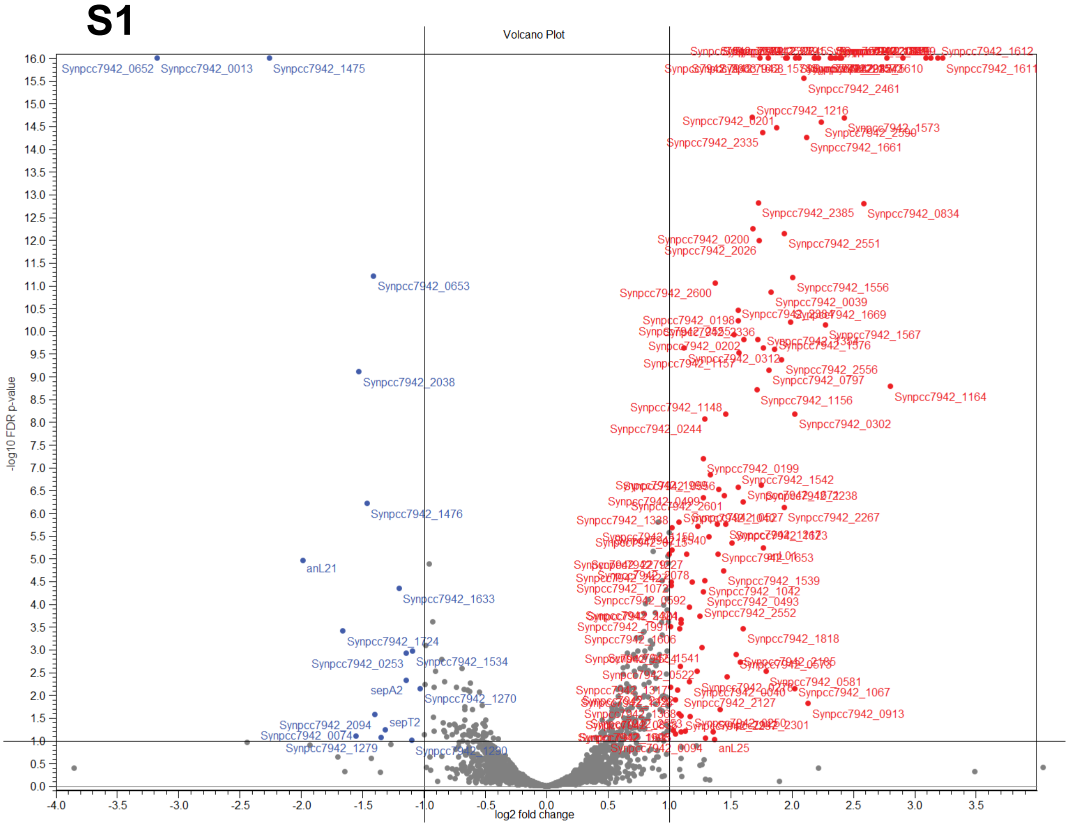

Supplementary Figure S2. Genes exhibiting expression changes in FDBR-expressing strains

Enlarged image of volcano plot shown in Figure 6A. Results of the comparison between WT and FDBR-expressing strains (AB1, X1, and S1). Upregulated and downregulated genes are depicted in red and blue, respectively ($\log_2$ FC ≥ ｜1｜, FDR $p \leq 0.1$). The X-axis corresponds to $\log^{2}$ fold change, and the Y-axis displays $-\log^{10}$ of an adjusted p-value of FDR. ORF IDs showing significant variation were indicated.

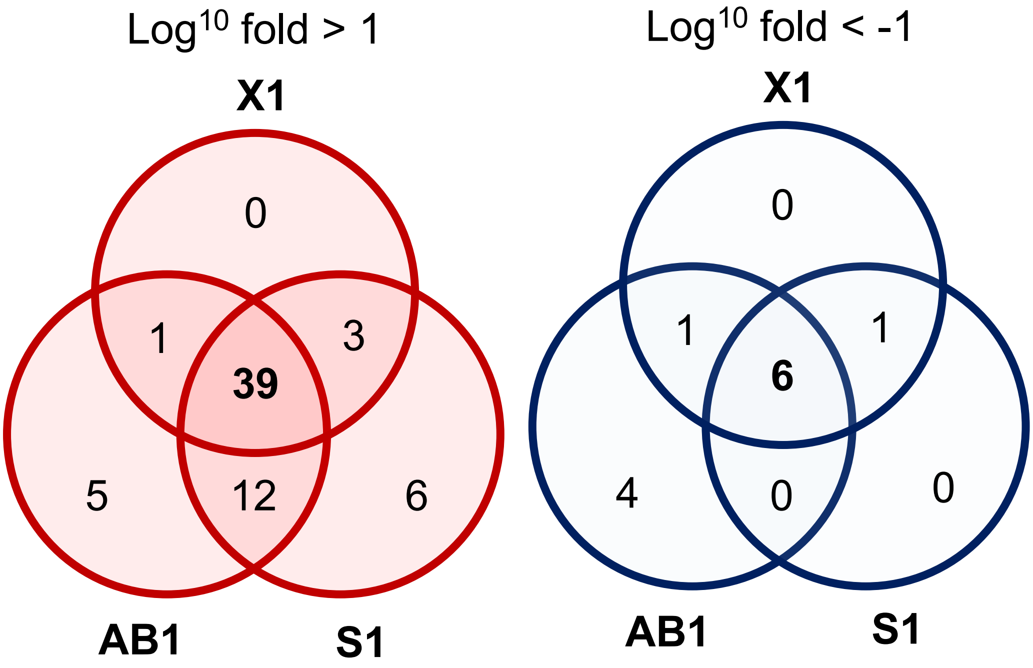

Supplementary Figure S3. Comparative transcriptomic analysis of Synechococcus elongatus PCC 7942 expressing different bilin reductases.

Venn diagram showing the overlap of differentially expressed genes (CDS) between wild-type (WT) cells and strains expressing *pebA-pebB* (AB1), *pcyX* (X1), or *pebS* (S1). CDSs were defined as differentially expressed when the absolute log^2 fold change exceeded 1 (i.e., log^2 fold change > 1 for up-regulated genes shown in red or < –1 for down-regulated genes in blue) and the p-values of false discovery rate (FDR) was < 0.1. The numbers in each section indicate the count of genes that are uniquely or commonly up- or down-regulated among the respective comparisons. The shared DEGs represent genes whose expression consistently responded to altered bilin biosynthesis across different enzyme introductions.

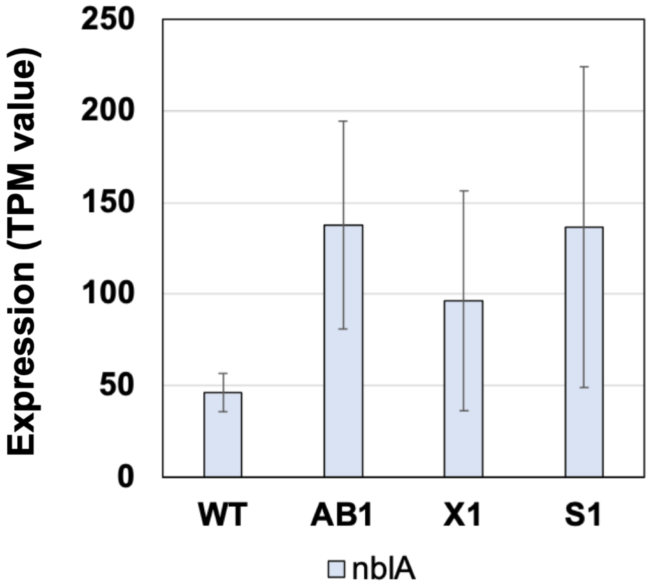

Supplementary Figure S4. The expression levels of *nblA* (Synpcc7942_2127) compared to the wild-type strain.

Data were extracted from Supplementary Table S1 and presented in the graph.

3. Table

Table S1. Comparison of transcriptome between WT and FDBRs expressing strains (additional Excel file)

Compared to WT, genes that increased by more than two-fold were highlighted in red, while those that decreased to less than half were highlighted in blue. 39 upregulated and 9 downregulated genes common to all three lineages ($\log_2 FC \geq |1|$, FDR $p \leq 0.1$) were extracted from the entire list.

Table S2. Oligonucleotide primers used for strain construction.

Primer name	Sequence (5′ to 3′)[a]
F1	GGAAACAGACCATGGAATTCATGATATGGGAACGCCTGATCA
R2	CCGCCAAAACAGCCAAGCTTTTACTTAATGGTGGGAAACAAGTTATC
F3	GGAAACAGACCATGGAATTCATGACAAAGAATCCTAGGAACAATAAACC
R4	CCGCCAAAACAGCCAAGCTTTTATTTGTAGCTGAATAAGAAGTC
F5	CATGGTCTGTTTCCTGTGTGAAATT
R6	AAGCTTGGCTGTTTTGGCGGATGAGAGAA
F7	CAGACCATGGAATTCTTCGACTCTTTCCTCAAC
R8	AAAACAGCCAAGCTTCTACGACAAGGAAGTTGAG
F9	CATCGCTATCTCTTAGGACTTCGCAG
R10	CAAACAGGTGCAGCAGCAACT

Table S3. Sequences of codon-optimized *pcyX* and *pebS* gene.

pcyX

ATGATATGGGAACGCCTGATCAAACTCGAGAAAGAGATCATTGAGGTTCTCGACAAACA
CCTGATTGAGTACAATGAGCCAGGAATGGATCGCTTCAACAAACCAGGATGGACGAATC
GGACATGGAGCAACATGTCCATACGTCGAGCACATGTGGATGTAGTCGATGCTCGAGAA
ACGAAAGGGTTATGGATGGCGCATATCTGCCTTTTCCCCATGAAGAAAAATGGTGGACC
GATTTATGGCTTTGACATAATTGCAGGCAAGAAGAAGGTTACTGGTGCGTTTCACGACTT
TTCTCCTCTGTTGAAAAAGGAGCATCCGTTAACTCGTTGGTTCATCGAAGAGAACAAGTG
GTACAAACCGTCAAAAGTACGCGAATTACCTGATTGGGCTAAAGCCATTTTCAGCGAAG
GCATGATTGCTGCAGGGAATGTGCAAGAGGAGAGGGAACTCAACCAAATCTGTACCATG
GCAGTCTCGAATCTTAACGCGTATATCGACAAAATCGGTCACTTCAATTCGGATTCCAAT
GAAGAGGATGTGATTCGTGCGCAAAACTTCTATTGCGAAAACCAGCAGAAAAATCCACA
TACACCTAGAACGATGAAGAGTCTAGGTTTGCCAGAAGAAGACATCAAACTGTTTTGTG
CCGATAACTTGTTTCCCACCATTAAGTAA

pebS

ATGACAAAGAATCCTAGGAACAATAAACCCAAAAAGATCCTTGATAGCTCGTATAAGTC
TAAGACGATTTGGCAGAACTACATTGATGCCCTATTTGAGACTTTTCCGCAACTTGAGAT
TTCAGAGGTTTGGGCTAAATGGGATGGTGGCAATGTCACGAAAGATGGTGGAGATGCGA
AATTGACAGCGAATATACGCACTGGCGAACACTTCTTGAAGGCTCGTGAAGCACATATC
GTGGACCCAAATTCCGACATCTACAACACGATTCTGTATCCGAAAACTGGTGCAGATCTC
CCCTGTTTTGGGATGGATCTCATGAAATTCTCAGACAAGAAAGTGATCATTGTCTTTGAC
TTTCAACATCCACGAGAGAAATACCTGTTTTCGGTAGACGGTTTACCGGAAGATGACGGC
AAATATCGCTTCTTCGAAATGGGGAATCACTTCTCTAAGAACATCTTTGTTCGGTATTGC
AAACCTGATGAAGTGGATCAGTATCTCGATACATTCAAACTGTATTTGACCAAGTACAAA
GAGATGATAGACAACAACAAACCTGTAGGAGAAGACACCACAGTTTACTCCGATTTTGA
CACCTATATGACGGAGTTAGATCCAGTAAGAGGTTATATGAAAAACAAGTTTGGCGAAG
GACGTAGTGAGGCATTCGTCAATGACTTCTTATTCAGCTACAAATAA

4. Gel images

The complete gel images before cropping were shown

Figure 5A

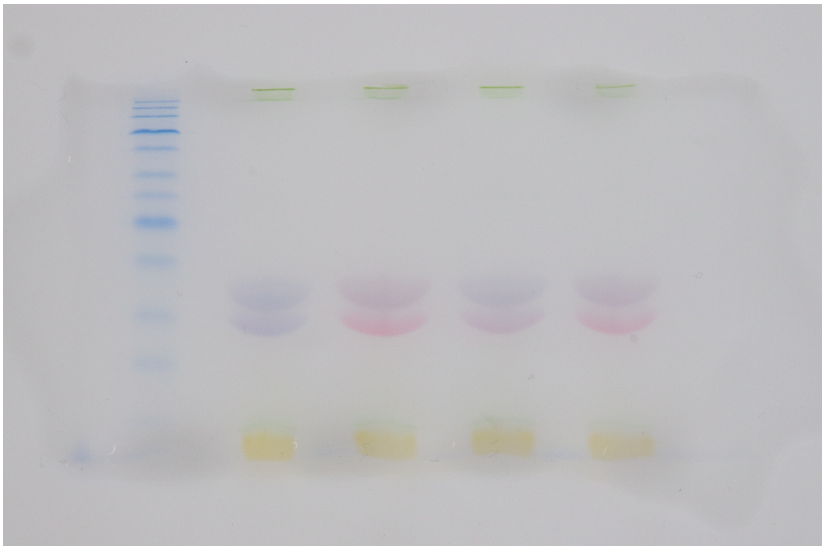

Supplementary Figure S1A

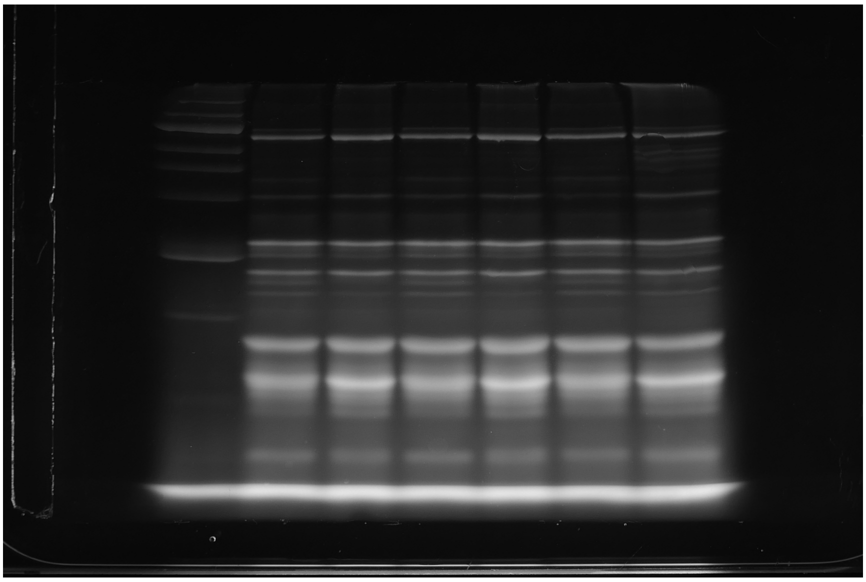

Supplement: Supplementary file 1 — Supplementary Material 1 [file 41598_2026_50582_MOESM1_ESM.pdf]
